# Hepatitis C Virus p7 Protein Is Crucial for Assembly and Release of Infectious Virions

**DOI:** 10.1371/journal.ppat.0030103

**Published:** 2007-07-20

**Authors:** Eike Steinmann, Francois Penin, Stephanie Kallis, Arvind H Patel, Ralf Bartenschlager, Thomas Pietschmann

**Affiliations:** 1 Department of Molecular Virology, University of Heidelberg, Heidelberg, Germany; 2 Institut de Biologie et Chimie des Protéines, UMR 5086 CNRS, Université Lyon 1, IFR 128 BioSciences Lyon-Gerland, Lyon, France; 3 Medical Research Council Virology Unit, Institute of Virology, University of Glasgow, Glasgow, United Kingdom; University of North Carolina, United States of America

## Abstract

Hepatitis C virus (HCV) infection is associated with chronic liver disease and currently affects about 3% of the world population. Although much has been learned about the function of individual viral proteins, the role of the HCV p7 protein in virus replication is not known. Recent data, however, suggest that it forms ion channels that may be targeted by antiviral compounds. Moreover, this protein was shown to be essential for infectivity in chimpanzee. Employing the novel HCV infection system and using a genetic approach to investigate the function of p7 in the viral replication cycle, we find that this protein is essential for efficient assembly and release of infectious virions across divergent virus strains. We show that p7 promotes virus particle production in a genotype-specific manner most likely due to interactions with other viral factors. Virus entry, on the other hand, is largely independent of p7, as the specific infectivity of released virions with a defect in p7 was not affected. Together, these observations indicate that p7 is primarily involved in the late phase of the HCV replication cycle. Finally, we note that p7 variants from different isolates deviate substantially in their capacity to promote virus production, suggesting that p7 is an important virulence factor that may modulate fitness and in turn virus persistence and pathogenesis.

## Author Summary

The hepatitis C virus (HCV), a major human pathogen associated with severe liver disease, encodes a small membrane protein designated p7. Although recent reports indicated that p7 forms channels conducting ions across membranes and is essential for HCV infection, its exact role in the viral life cycle remained elusive. In this study, we illustrate that HCV relies on p7 function for efficient assembly and release of infectious progeny virions from liver cells. Conversely, entry of HCV particles into new host cells is independent of p7. This new evidence supports the recent proposal to include p7 into the family of viroporins that comprises proteins from diverse viruses, for instance, HIV-1 and influenza A virus. Members of this group of functionally related proteins form membrane pores that promote virus release and in some cases also virus entry. Moreover, we identify several conserved p7 residues crucial for functioning of this protein. These amino acids possibly stabilize the structure of p7 or directly participate in channelling of ions. Interestingly, p7 variants from divergent patient isolates differ with regard to their ability to promote virus production, suggesting that p7 modulates viral fitness. Together these observations shed new light on fundamental aspects of the HCV replication strategy.

## Introduction

Hepatitis C virus (HCV) is an enveloped virus that presently has chronically infected about 170 million people worldwide. One hallmark of HCV is its high degree of sequence variability that likely contributes to its ability to establish chronic infections. Different patient isolates are grouped into six genotypes (GTs) and more than 100 subtypes within the genus *Hepacivirinae* of the family Flaviviridae [1]. Persistent infection is associated with a variable degree of liver damage often progressing in severity over the course of decades. Accordingly, a large number of patients are at risk of severe sequelae, including life-threatening conditions like cirrhosis and hepatocellular carcinoma [2]. The best available treatment, a combination of polyethylene glycol-conjugated interferon alpha and ribavirin, is effective in only a fraction of patients and is associated with severe side effects (reviewed in [3]). A prophylactic or therapeutic vaccine is not available.

**Figure 1:**
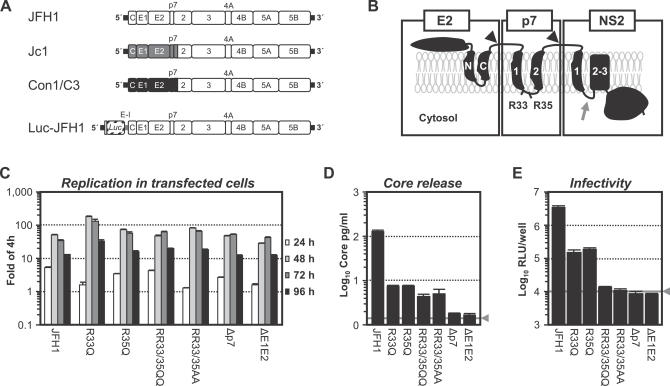
Replication and Virus Production of Luc-JFH1 Genomes with Mutated p7 (A) Schematic representation of constructs used in this study. JFH1-derived 5′ and 3′ nontranslated regions are drawn as thick black lines and JFH1 proteins are depicted as open boxes. Jc1 and Con1/C3 have been described and comprise chimeric HCV polyproteins consisting of J6CF (gray boxes) or Con1 (black boxes) fused with JFH1 [19]. The luciferase reporter virus genome Luc-JFH1 is depicted at the bottom [21]. (B) Topology model of E2, p7, and NS2 proteins. Signalase cleavage sites are denoted by black arrowheads. The location of conserved di-basic motif of p7 is indicated. C- and N-terminal portion of the E2 TM domain are labeled N and C, respectively, while predicted TM helices 1 and 2 of p7 are labeled 1 and 2. Predicted NS2 TM helix 1 is labeled 1 while the two following predicted helices are labeled 2/3. Note that the crossover site in the virus chimeras is located in the loop following TM helix 1 of NS2 (gray arrow) [19]. (C) Replication of Luc-JFH1 and given mutants in transfected Huh7-Lunet cells determined by luciferase reporter assays. Values given are expressed relative to the reporter activity measured at 4 h which was set to one. Mean values of duplicate measurements and the standard errors are given. (D) Release of core protein into the culture fluid 72 h post-transfection of cell given in (C). The gray lines indicate the cut-off of the ELISA and the background luciferase activity measured in mock-infected cells, respectively; mean values of duplicate measurements and the standard errors are given. (E) Infectivity associated with culture fluids harvested 72 h post-transfection determined by inoculation of Huh-7.5 cells and reporter assays. Mean values of duplicate wells and the standard error of the means are given. The gray line denotes background relative light units measured in mock-infected cells. Data presented in (C–E) were derived from a single experiment representative of five independent repetitions.

HCV possesses a positive strand RNA genome of about 9.6 kb composed of nontranslated regions at the 5′ and 3′ termini required for translation and RNA replication and a single open reading frame encoding a large polyprotein (for a recent review see [4,5]) (Figure 1A). A set of processing events mediated by cellular enzymes and two viral proteases, NS2–3 and NS3-4A, liberate the individual viral protein core, envelope glycoproteins 1 and 2 (E1, E2), p7, and nonstructural proteins NS2, NS3, NS4A, NS4B, NS5A, and NS5B.

Specific functions have been ascribed to most viral proteins: core, E1, and E2 are the structural constituents of the virus particle, and the viral genome is amplified by a membrane-associated replicase complex consisting of nonstructural proteins NS3–NS5B; the role of the small membrane polypeptide p7, however, is not defined. While recent in vivo experiments clearly indicate that p7 is essential for infection [6], subgenomic HCV replicons do not contain p7, demonstrating that it is not necessary for RNA replication [7]. Ectopically expressed, p7 primarily localized to the endoplasmic reticulum as an integral membrane protein and displayed a topology with both N- and C-termini pointing toward the endoplasmic reticulum lumen [8]. In line with this, structure predictions suggest that p7 comprises two transmembrane (TM) helices connected via a short cytoplasmic loop (Figure 1B) [8,9]. Recent in vitro studies indicate that p7 oligomerizes [10,11] and is capable of conducting ions across artificial membrane systems in a cation-selective manner [10,12,13]. Ion channel activity has been reported to be specifically blocked by various compounds, suggesting that this function may be amenable for development of antiviral therapy [10,12,13]. Due to these properties, p7 has been tentatively included in the family of viroporins comprising proteins from diverse virus families, as for instance, 6k of alpha-viruses, M2 of influenza virus A, or Vpu of HIV-1. Viroporins are generally small polypeptides that oligomerize, thus forming hydrophilic pores that modulate membrane permeability to ions in a way that is conducive for virus propagation by promoting virus release, and in the case of M2, also virus entry [14]. In this study, we used a genetic approach to investigate the biological function of p7 in the replication cycle of HCV. Our data indicate that this protein is crucial for a late step in virus assembly and release, thus providing functional evidence that p7 acts as a classical viroporin in the course of HCV replication.

## Results

### p7 Is Required for Production of Infectious JFH1 Particles

Poor replication of HCV in cultured cells has long hampered analysis of the virus life cycle. Only recently, this impediment was overcome by the development of HCV infection systems based on the JFH1 strain (GT 2a) or a cell culture–adapted variant of the H77 isolate [15–18]. The scope of the former system was extended by construction of chimeras consisting of JFH1-derived replicase proteins and structural proteins from heterologous isolates, allowing comparative analyses of assembly and infection (Figure 1A) [15,19]. In addition, insertion of reporter genes (e.g., luciferase) into these constructs (Figure 1A) now allows for a fast and accurate measurement of replication and infection [16,20,21].

To study the function of p7, we utilized the Luc-JFH1 reporter viruses, introduced mutations into the coding region of p7, and assessed their impact with respect to RNA replication and release of infectious virus particles. As reference, we used a variant genome carrying a large in-frame deletion in the E1-E2 coding region known to inactivate release of virus particles [16]. For comparison with previous observations, we replaced the two conserved basic residues R33 and R35 located in the predicted cytoplasmic loop of JFH1-derived p7 by alanine. These residues are known to be essential both for in vivo infectivity [6] and in vitro ion channel activity of p7 [22]. In addition, we mutated these positions either individually or simultaneously to glutamine, which due to its polarity is not expected to disturb the overall topology of p7. Finally, we created a mutant lacking residues 1–32 of p7, directly connecting the C-terminal half of p7 carrying a signal-like sequence [8] to the C-terminus of E2 (Figure 1B). None of these alterations affected RNA replication in transfected Huh-7 cells (Figure 1C). In contrast, all mutations influenced the number of infectious particles released as indicated by lower amounts of core protein in the culture fluid (Figure 1D) and a decreased or abolished transduction of luciferase reporter activity (Figure 1E). While mutation of either R33 or R35 to glutamine resulted in a partial defect, for all other mutants release of infectious particles was no longer detectable, indicating that p7 is required for the assembly and/or release of infectious virions.

### Identification of Conserved Amino Acids Involved in the Function of p7

**Figure 2:**
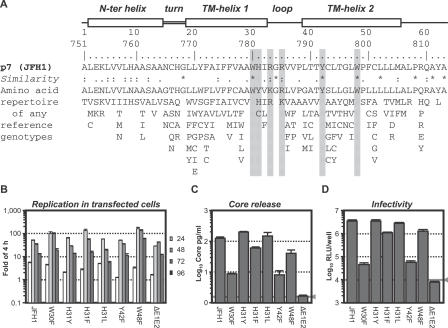
Identification of Conserved Amino Acids Essential for p7 Functioning (A) Amino acid repertoire of the 26 representative p7 sequences of confirmed HCV genotypes/subtypes (listed with accession numbers in Table 1 in [1]). Helical turn and loop regions deduced from the nuclear magnetic resonance structure analysis of a p7 GT 1b variant (F. Penin, unpublished data) are depicted at the top, and the primary sequence of JFH1-derived p7 (GT 2a) in combination with a ruler indicating amino acid numbers with respect to p7 and the complete JFH1 genome are given below. The degree of conservation can be inferred by the extent of variability (with the observed amino acids listed in decreasing order of frequency from top to bottom) and the similarity index according to ClustalW convention (asterisk, invariant; colon, highly similar; dot, similar) [46]. Residues mutated in this study are shaded in gray. (B) Replication of Luc-JFH1 and given mutants in transfected Huh7-Lunet cells determined as described above. (C) Release of core protein and (D) infectivity into the culture fluid 72 h post-transfection of cell given in (B). The gray lines indicate the cut-off of the ELISA and the background luciferase activity measured in mock-infected cells, respectively. A representative experiment of at least four independent repetitions is given.

To identify conserved amino acids likely essential for the structure and/or function of p7, we investigated the variability of p7 by using a ClustalW alignment of 26 reference genotypes representative of all major genotypes and subtypes [1]. Based on this alignment we derived the amino acid repertoire given in Figure 2A that lists the various amino acids observed at every position of the p7 primary sequence in decreasing order of frequency. In total, nine invariant amino acids (labeled by an asterisk) emerged, among these three cyclic amino acids. As cyclic residues, like histidine, tyrosine and tryptophan are essential for the structure-function relationships of other viral ion channel proteins (e.g., M2 of influenza A virus [23] or Vpu of HIV-1 [24]); therefore, we focused on fully conserved W30, Y42, and W48, and in addition, on the more variable residue H31. To minimize effects on topology and folding of p7, we substituted these residues with phenylalanine, as this alteration should conserve the predicted alpha-helical folding and respect the cyclic character of these residues. In contrast, phenylalanine is not expected to substitute for a direct involvement of the given residue in ion channelling. In case of H31, we created a set of mutants (H31Y, H31F, and H31L) that are expected to have increasing impact if this residue directly participates in ion channelling.

None of these alterations affected RNA replication in transfected Huh-7 cells (Figure 2B). In contrast, release of core protein (Figure 2C) and infectivity (Figure 2D) was severely impaired for W30F and Y42F mutants but unchanged or only slightly affected for all other mutants, suggesting that W30 and Y42 are involved in the structure-function relationship of p7.

**Figure 3:**
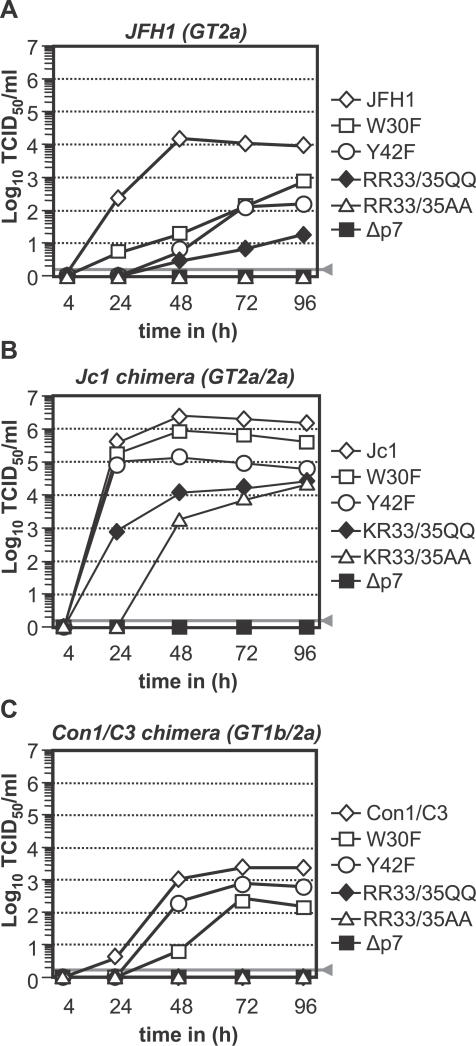
Accumulation of Infectivity upon Transfection of JFH1 or Chimeras Carrying Varying p7 Mutations Huh-7.5 cells were transfected with (A) JFH1, (B) Jc1, or (C) Con1/C3 wild-type constructs or given p7 mutants in the context of the respective virus. Cell-free supernatants were harvested and titrated by using a limiting dilution assay. The gray bars represent the detection limit of the limiting dilution assay.

Next, we investigated if these residues are important across different HCV genotypes. To this end we introduced these mutations into JFH1, the Jc1, and the Con1/C3 chimera, the latter constructs comprising core, E1, E2, p7, and the amino terminal portion of NS2 derived from J6CF (GT 2a) or Con1 (GT 1b), respectively [19]. As noted previously [19], JFH1, Jc1, and Con1/C3 differ with respect to extent and kinetics of accumulation of infectious virions (Figure 3A–3C, respectively). Nonetheless, the impact of p7 mutations was remarkably congruent. In all three viruses, the introduced p7 mutation did not affect RNA replication, as evidenced by comparable intracellular core protein expression 24 or 48 h post-transfection (unpublished data). Conversely, partial deletion of p7 abrogated production of infectious particles (Figure 3A–3C), as did double mutation of basic amino acids to alanine at position 33 and 35 in the event of JFH1 and Con1/C3. In case of Jc1, the latter mutation delayed the kinetics of virus accumulation and reduced the peak titer attained almost 100-fold. Mutation of these residues to glutamine also exerted a severe effect as accumulation of infectivity was no longer detectable for Con1/C3, very low for JFH1, and both delayed and reduced in the event of Jc1. Finally, mutation of conserved W30 or Y42 residues decreased the amount of infectivity produced for all three viruses, albeit to a lesser extent than the mutants described above.

**Figure 4:**
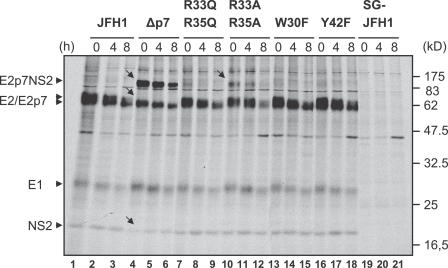
Processing of the E2-p7-NS2 Region of JFH1 and Various p7 Mutants Huh-7.5 cells were transfected with indicated constructs and after 24 h pulse-labeled with [35S] methionine/cysteine-containing medium, lysed immediately, or chased for 4 or 8 h. Cells transfected with a subgenomic JFH1 replicon encoding HCV proteins NS3–NS5B only (SG-JFH1) served as negative control. HCV proteins were immunoprecipitated using an antibody monospecific for E2 and are identified by arrows on the left. The positions of the molecular weight standard are given on the right. Arrowheads to the left point to the respective precursors and mature proteins detected. Arrows within the figure point out obvious alterations in protein processing.

It is worth noting that some mutations of p7 completely blocked release of infectious particles while others exerted a more or less pronounced defect, suggesting that either the function of p7 was not completely inactivated by the latter, or that the former mutants caused additional defects impacting on virus production (e.g., signalase cleavage events at the E2-p7 and/or p7-NS2 site). To distinguish between these possibilities, we analyzed the processing of E2, p7, and NS2 by pulse-labeling of Huh-7.5 cells transfected with JFH1 or the different JFH1-p7 mutants and immunoprecipitations using an E2-specific monoclonal antibody (Figure 4). For all p7 mutants, similar amounts of E2, E1, and NS2 were precipitated when compared to JFH1, indicating that processing at the E2-p7 and p7-NS2 junction occurs and yields similar quantities of E1-E2 complexes. However, mutant RR33/35AA, and in particular Δp7, displayed clearly more unprocessed E2-p7-NS2 precursor compared to JFH1 and to all other mutants, indicating that signalase cleavage was impaired by these mutations. Moreover, in case of Δp7, no E2-p7 species could be detected and slightly less NS2 was precipitated. Therefore, for RR33/35AA and Δp7 mutants, we cannot rule out that the complete blockage of virus production was at least in part caused by a defect of processing steps. However, in case of mutants RR33/35QQ, W30F, and Y42F with the resolution of the employed assay, processing was not affected, suggesting that impaired virus production in these mutants is likely specifically due to interference with the function of p7. In conclusion, these data demonstrate that conserved amino acids R33/35, W30, and Y42 are key residues involved in the functioning of p7 across different HCV genotypes.

### p7 Is Involved in the Assembly and Release of HCV Particles

**Figure 5:**
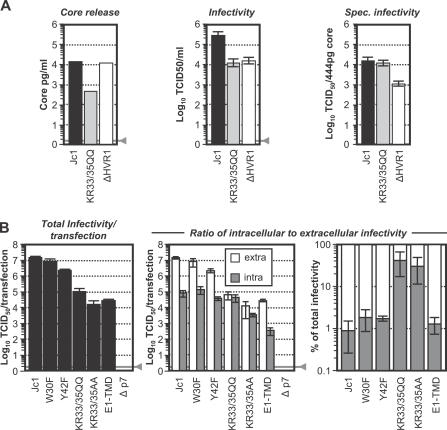
Specific Infectivity and Efficiency of Virus Assembly and Release of Jc1 with Mutated p7 (A) Huh7-Lunet cells were transfected with indicated genomes, and 48 h post-transfection culture fluid was harvested for quantification of core protein by ELISA (left panel) and infectivity by TCID_50_ assay (middle). The specific infectivity of the given viruses was determined by dilution of Jc1 and ΔHVR1 to the same quantity of core protein as KR33/35QQ (444 pg) and titration by using the limiting dilution assay. (B) Cells were transfected as in (A) and 48 h post-transfection supernatants were collected. In parallel, virus-producing cells were washed and lysed by repetitive cycles of freeze and thaw. Extracellular (white bars) and intracellular infectivity (gray bars) were determined by limiting dilution assay and are depicted in the middle. The panel on the left shows the total infectivity per plate, whereas the percentage of cell-associated and released infectivity expressed relative to the total amount of infectivity is given in the right. The gray lines denote the detection limits of the ELISA and the limiting dilution assay, respectively. Mean values of five independent experiments and the standard deviation of the means are presented.

The M2 ion channel of influenza A virus is incorporated into virus particles and is probably involved both in virus assembly and entry (reviewed in [25]). Due to lack of antibodies and difficulties in producing sufficient virus, it is not known if p7 is a constituent of HCV virions. Moreover, it is unclear if p7 participates in virus entry. If this was the case, viruses with mutant p7 should be impaired in entry and thus exhibit lower specific infectivity (i.e., infectivity per given number of physical particles). Therefore, we compared particle release, accumulation of infectivity, and the specific infectivity of particles between Jc1, the Jc1 p7 mutant KR33/35QQ, and Jc1ΔHVR1, a derivative carrying a deletion of the hyper-variable region 1 (HVR1) of E2, a domain known to be involved in receptor interactions [26,27] (Figure 5A). The p7 mutation (KR33/35QQ) suppressed release of particles, whereas deletion of HVR1 did not affect particle release (Figure 5A, left). Both mutants, however, displayed impaired infectivity, likely due to disturbed receptor interaction, and thus diminished specific infectivity in the case of Jc1ΔHVR1 and lower numbers of released particles for the KR33/35QQ mutant (Figure 5A, middle). To rigorously assess if the latter mutant is additionally impaired in virus entry, we diluted the Jc1 and Jc1ΔHVR1 culture fluids to the same quantity of core protein as present in the Jc1-KR33/35QQ preparation and titrated the infectivity associated with this number of physical particles (i.e., specific infectivity, Figure 5A, right). While Jc1ΔHVR1 particles were about 10-fold less infectious than Jc1 particles, the KR33/35QQ mutant was indistinguishable from Jc1, indicating that this p7 mutation did not affect the capacity of these particles to enter Huh-7 cells. It is of note that these results are in agreement with the data obtained with p7 mutants in the context of Luc-JFH1 reporter viruses (Figures 1 and 2). In this setting, mutations R33Q, R35Q, W30F, and Y42F impaired production of infectious particles and release of core protein in a commensurate manner, indicating that the specific infectivity of these virions was not affected by the respective mutations in p7.

The data presented so far indicate that mutation of p7 decreases the number of secreted infectious particles. This may be attributable to defective virion assembly or impaired release of virions. To distinguish between these possibilities, we compared the quantity of intracellular infectious viruses (prepared by repetitive cycles of freeze and thaw [28]) and extracellular infectious viruses produced upon transfection of Huh-7 cells with Jc1 and the p7 mutants W30F, Y42F, KR33/35QQ, KR33/35AA, and Δp7 (Figure 5B). As reference, we constructed a Jc1 mutant encoding glutamine in place of the conserved lysine in the TM domain of E1 (K179Q; designated E1-TMD). Since K179 of E1 is involved in heterodimerization between E1 and E2 [29], likely a prerequisite for virus formation, we assumed that its mutation should primarily affect assembly of infectious virions. Figure 5B displays the total infectivity (summation of extracellular and cell-associated infectivity) produced upon transfection of these constructs (left), the quantity of cell-associated and extracellular infectivity (middle), and where applicable, the percentage of cell-associated and released infectivity expressed relative to the total amount of infectivity (right).

As expected, p7 mutations W30F and Y42F reduced total infectivity by 2- and 5-fold only, while for mutants KR33/35QQ and KR33/35AA, and the E1 mutant E1-TMD, total yields of infectious virions were heavily impaired (∼100-, 1,000-, and 500-fold, respectively). In case of E1-TMD the numbers of intra- and extracellular infectious virions were decreased in a commensurate manner (∼500-fold) when compared to Jc1, indicating that this mutation affected assembly and not the efficiency of virus release. Accordingly, the ratio of intracellular to extracellular infectivity was indistinguishable from Jc1 with much more infectious particles liberated into the culture fluid (∼99%) than retained intracellularly (Figure 5B, right). In contrast, mutation of KR33/35 to either QQ or AA primarily decreased the quantity of infectious virions released from the cells. As a consequence, these mutants displayed a much higher proportion of cell-associated particles when compared to Jc1 and Jc1-E1-TMD (∼30%–40% as compared to 1%–2%) indicating that these p7 mutants were impaired both in assembly and release of infectious viruses. For mutants W30F and Y42F, which produced only slightly or moderately less infectious virions, we did not observe a significantly changed ratio between extra- and intracellular virions. Finally, Jc1Δp7 did not produce any infectivity, indicating that this mutation is lethal to proper virus production. In summary, these data indicate that p7 is not only essential for efficient assembly of infectious virus but also critical for effective export of infectious particles.

### p7 Variants Differ with Regard to Their Ability to Promote Virus Production

**Figure 6:**
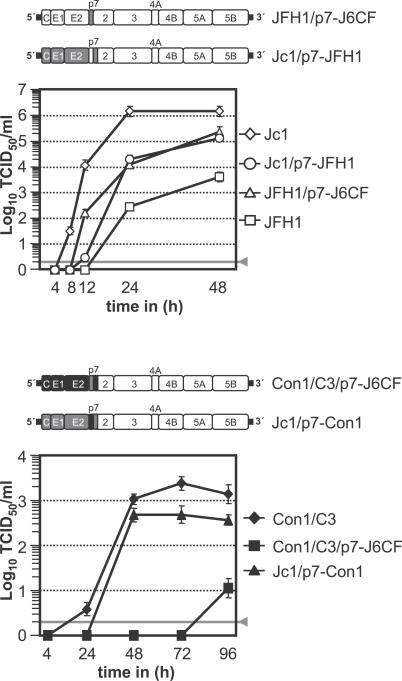
Accumulation of Infectivity upon Transfection of GT 2a and GT 1b Viruses with J6CF-Derived p7 Proteins The color coding is as in Figure 1 with open boxes representing JFH1, gray boxes J6CF, and black boxes Con1 proteins. Accumulation of infectivity upon transfection is given below. The gray line denotes the detection limit of the limiting dilution assay. A representative experiment of at least two repetitions is depicted.

Why HCV chimeras with J6CF-derived core-to-NS2 proteins in place of the homologous JFH1 proteins produce much higher virus titers is unknown. Based on the observation that p7 is a key determinant of efficient assembly and virus egress, we speculated that this may be linked to the J6CF-derived p7. Thus, we constructed additional chimeras by transferring J6CF-derived p7 to JFH1 or Con1/C3 (Figure 6). Moreover, in the Jc1 backbone, we substituted J6CF p7 for the one of JFH1 or Con1. In line with the above hypothesis, interchange of p7 proteins profoundly altered the efficiency of virus production of the different virus chimeras while not affecting RNA replication, as evidenced by comparable intracellular core protein expression 24 h post-transfection (unpublished data). In case of intragenotypic GT 2a chimeras (JFH1 and J6CF), J6CF p7 conferred much better growth properties (Figure 6, top). More specifically, transfer of this p7 variant into JFH1 increased the peak titer more than 20-fold over JFH1. In contrast, incorporation of JFH1 p7 into the context of Jc1 delayed accumulation of infectivity and reduced the peak titer about 10-fold compared to Jc1. Thus, for these GT 2a chimeras, properties inherent to J6CF p7 bestow much more rapid and efficient virus production. However, this attribute of J6CF p7 does not promote virus production independent of virus strain as is evident from the intergenotypic transfer of J6CF p7 to Con1/C3 (GT 1b) which suppresses virus production (Figure 6, bottom). In this case, likely genetic incompatibility between GT 2a p7 and GT 1b structural proteins or the N-terminal NS2 domain of GT 1b limits virus production.

**Figure 7:**
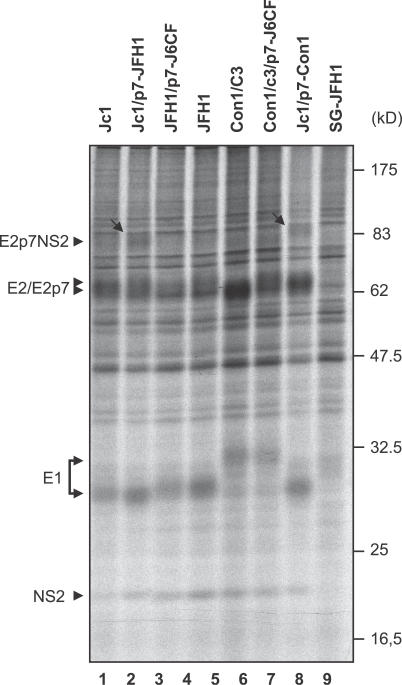
Processing of the E2-p7-NS2 Region of JFH1, Jc1, Con1/C3, and Various Chimeras Huh-7.5 cells were transfected with indicated constructs. 24 h post-transfection, cells were labeled with [35S] methionine/cysteine-containing medium overnight and then lysed immediately. HCV proteins were immunoprecipitated using an antibody monospecific for E2. The positions of the molecular weight standard are given on the right. Arrowheads to the left point to the respective precursors and mature HCV proteins detected. Arrows within the figure point out alterations in protein processing. Note that Con1-derived E1 displays a lower electrophoretic mobility than E1 from JFH1 and J6CF.

Beside this, we observed subtle changes at the level of processing in the E2-p7-NS2 region in case of the Jc1/p7-JFH1 and Jc1/p7-Con1 chimeras that carry JFH1-derived and Con1-derived p7 in place of the autologous J6CF p7, respectively (Figure 7; compare lane 1 with lanes 2 and 8). Finally, when transferring J6CF-p7 to the Con1/C3 chimera we noted a minor alteration of the E2/E2p7 signal that may indicate a change of processing at the E2-p7 junction or an altered glycosylation of these proteins (Figure 7, lanes 6 and 7). This modified processing may, in addition to genetic incompatibility, limit production of infectious virions, thus complicating direct comparison of the ability of a given p7 variant to promote virus production. Nevertheless, at least within a given genotype, the pronounced gain-of-function when exchanging JFH1 p7 for the one encoded by J6CF clearly indicates that p7 from divergent virus strains possesses different capabilities to facilitate virus production, and that J6CF-p7 is more active in this regard than JFH1-p7.

## Discussion

Accumulating evidence indicates that p7 is capable of forming ion channels [10,12,13]. Based on this property and its predicted structure and topology [8,9] it was ascribed to the family of viroporins. By definition, these are small, rather hydrophobic viral proteins which tend to oligomerize to form hydrophilic pores that modulate membrane permeability conducive for virus propagation [14]. Currently, a number of proteins from divergent viruses are assigned to this family, among them prominent examples like influenza A virus M2, HIV-1 Vpu, and 6k of alpha-viruses. While M2 facilitates both virus entry and exit, Vpu and 6k only assist virus assembly and release. Although not absolutely essential, they greatly facilitate these steps in the viral replication cycle. Accordingly, inactivation of 6k or Vpu diminishes yields of infectious particles in the culture fluid and increases the quantity of cell-associated particles [30–34].

By using a set of viruses carrying specific p7 mutations, we found that this protein is involved in assembly and release of infectious virus particles; thus providing strong functional evidence that p7 acts as classical viroporin in the replication cycle of HCV. This conclusion rests on the lower yields of infectious particles that we obtained upon transfection of viruses with several different p7 mutations. Moreover, when p7 was mutated we noted a much higher ratio of cell-associated to free virus particles as compared to Jc1 or a mutant with impaired glycoprotein heterodimerization. This is reminiscent of the phenotype caused by 6k or Vpu mutation and thus indicates that p7, like Vpu and 6k, acts at a very late step of virus assembly, possibly facilitating virus budding.

The specific infectivity of viruses with mutant p7, however, was not reduced, suggesting that p7 does not play a role during virus entry. Although we cannot exclude that the mutations analyzed may not affect a putative function of p7 in virus entry, this is unlikely as several mutations in different regions of the protein impaired virus release but did not change the specific infectivity of released particles (compare Figures 1, 2, and 4). It is worth mentioning that based on these data it is unlikely that the introduced mutations act by inactivating *cis*-active RNA packaging signals as this would likely result in a decrease of specific infectivity of secreted virions due to impaired packaging of genomic RNA. In agreement with this notion, we have observed efficient *trans*-packaging of subgenomic HCV replicons (comprising the nontranslated regions and the coding region of NS3 to NS5B only) by full-length helper viruses, demonstrating that the core to NS2 region of the HCV genome does not carry *cis*-active RNA signals absolutely essential for genome packaging (E. Steinmann and T. Pietschmann, unpublished data).

Besides this, we have shown that replacement of the two conserved basic amino acids within the cytoplasmic loop of p7 with either alanine or glutamine dramatically suppressed production of infectious particles of GT 1b and GT 2a viruses. Interestingly, Sakai et al. found that a similar mutant (KR33/35IS) in the context of H77c (GT 1a) is not infectious in chimpanzee [6]. Moreover, Griffin et al. recently reported that p7 derived from the infectious molecular clone HC-J4 (GT 1b; [35]) substituted for M2 in a cell-based ion channel assay whereas the KR33/35AA mutant did not [22]. It is of note that in our pulse-labeling experiments of transfected Huh-7 cells producing HCVcc, we have observed a slight defect at the level of E2-p7-NS2 processing for the JFH1 p7 mutant RR33/35AA. Therefore, we cannot exclude that the dramatic effect of this mutation may at least in part be caused by aberrant processing events at the E2-p7 and p7-NS2 junction. In fact, mutation of the di-basic motif close to the cytoplasmic loop of p7 by hydrophobic ala residues is expected to disturb the signal-like function of the C-terminal half of p7, and also possibly p7 topology. Conversely, their mutation to hydrophilic Gln residues (RR33/35QQ) is not expected to exert these adverse effects, and consequently we did not detect a defect at the level of E2-p7-NS2 processing. Therefore, for the latter mutant the pronounced defect in virus production is likely primarily attributable to a loss of p7 function. In synopsis with our data, these results highlight the importance of the conserved di-basic motif at the C-terminus of the first TM helix of p7, and moreover, they provide a rational explanation why the mutant described by Sakai et al. was noninfectious in vivo. It is worth mentioning that our data are similar to those of Harada et al. who observed that p7 of the bovine viral diarrhea virus (BVDV), a relative of HCV belonging to the genus *Pestivirinae* within the family Flaviviridae, is required for production of infectious particles [36]. Despite little sequence similarity, the BVDV equivalent also contains two hydrophobic regions connected by a polar segment containing several conserved charged residues. Remarkably, mutation of these abrogated production of infectious BVDV particles [36]. This indicates that these proteins may adopt a similar topology and act as functional homologs and suggests that both viruses rely on viroporin function for efficient virus assembly and particle export.

To define further amino acids and protein domains involved in the function of p7, we performed an exhaustive analysis of sequence conservation based on reference sequences of all major genotypes. Site-directed mutagenesis revealed that W30 in close proximity to the di-basic motif and Y42 at the beginning of TM helix 2 are essential for p7 functioning across different genotypes. On the other hand, W48 and H31 appear to be less essential, as mutation of these residues did not significantly affect particle production. Without a high resolution structure of p7 it is difficult to predict how these changes influence p7 conformation and/or function. In principle, interactions between p7 TM-helices, p7 monomers, or other viral or cellular proteins may be affected. Alternatively, ion channelling, or possible gating mechanisms required for proper function may be impaired. Interestingly, when we mapped the residues analyzed in this study on a model of JFH1-derived p7, which is based on the secondary structure of a GT 1b p7 variant recently solved by nuclear magnetic resonance spectroscopy (Figure S1 and F. Penin et al., unpublished data), the side chains of W30 and Y42 interacted by the stacking of their aromatic rings directed toward each other at the interface between TM helix 1 and 2. In contrast, the side chains of the conserved basic amino acids K33 and R35 at the C-terminal end of TM helix 1, point away from the p7 monomer toward the negatively charged lipid head groups at the cytoplasmic face of the membrane. Therefore, this model structure indicates that W30 and Y42 interaction is essential for the stabilization of p7 TM helix packing, whereas the di-basic motif may be directly involved in ion channelling, as supported here by the glutamine mutants. Alternatively or in addition, these basic residues may stabilize a proper orientation of p7 with respect to the membrane via electrostatic interactions with negatively charged phosphate of lipid polar head groups.

Finally, it is interesting to note that the apparently highly efficient J6CF p7 variant boosted virus production in the context of the homologous GT 2a strain JFH1, but almost abrogated accumulation of infectious virions in the environment of the more distantly related GT 1b virus Con1/C3. On one hand, these data indicate that p7 variants promote virus assembly and release with rather different potency, suggesting that p7 significantly contributes to viral fitness and in turn may affect virus persistence and pathogenesis. On the other hand, based on these results we conclude that interactions of p7 with other viral structural proteins and/or NS2 are important for p7 to function in promoting virus assembly and release. This notion is supported by several lines of evidence: First, Haqshenas et al. recently reported that an intergenotypic JFH1 chimera with GT 1b p7 was viable yet attenuated when compared to JFH1 [37]. Second, Yi et al. described that an intergenotypic virus chimera comprising H77 and JFH1 segments fused at a position within NS2 acquired compensatory mutations residing in p7 and NS2 [38]. Third, when we mapped the optimal junction for Con1-JFH1 chimeras, a crossover downstream of the first TM-helix of NS2 was superior to a junction at the C-terminus of p7 [19]. And finally, Sakai et al. noted that an H77c chimera with J6CF p7 was only infectious in chimpanzee when the endoplasmic reticulum–resident N- and C-terminal loop regions were derived from H77 [6], suggesting that genotype-specific interactions may at least in part be mediated by these domains.

In summary, we show that HCV p7 promotes a late step of assembly and release of infectious virions. These data support the notion that p7 acts as viroporin likely similar to its BVDV equivalent, to alpha-virus 6k, and HIV-1 Vpu. In addition, we show that virus isolates differ in regard to their dependence on p7 function and the potency of the respective p7 variants to promote virus assembly and release. The stimulatory effect of p7 on virus production is controlled by the genotype of other viral proteins implying that genotype-specific interactions of p7 govern the efficiency of assembly. Finally, the knowledge of conserved residues involved in the function of p7 should help to evaluate in vitro assays useful for screening of p7-specific antiviral compounds and to further dissect the mechanism of HCV assembly.

## Materials and Methods

### Plasmids.

Plasmids pFK-JFH1, pFK-Jc1, pFK-Con1/C3, and pFK-Luc-JFH1 have been described recently [16,19]. To facilitate mutagenesis, we introduced a silent XbaI site at the beginning of the p7 coding region of each of these constructs. This modification did not affect HCV RNA replication or virus production (unpublished data). Individual mutations were introduced by standard PCR-based techniques and verified by sequencing. Detailed sequence information is available upon request.

### Cell culture and infectivity assay.

Huh-7 cell–derived cell clones Huh-7.5 and Huh7-Lunet, which are highly permissive for HCV replication [39,40], were used for transfection and infection assays. Luciferase reporter virus-associated infectivity was determined as described [21]. Authentic viruses were titrated by using a limiting dilution assay on Huh-7.5 cells [15] with a few minor modifications [41]. The tissue culture 50% infectivity dose (TCID_50_) was calculated based on the methods described [42,43].

### In vitro transcription, electroporation, and transient HCV replication assay using authentic and reporter genomes.

These methods have been described elsewhere [16,41]. When genomes without reporter gene were transfected, we controlled transfection and replication efficiency of these genomes by determining intracellular core protein expression 24 h or 48 h post-transfection.

### Freeze and thaw lysates of HCV-transfected cells.

Huh7-Lunet cells were transfected with parental and recombinant Jc1 genomes; 48 h post-transfection cell culture supernatants were harvested and virus titers determined by TCID_50_. Cell-associated infectivity was prepared essentially as described [28]. Briefly, cells were extensively washed with PBS, scraped and centrifuged for 5 min at 400 × *g*. Cell pellets were resuspended in 1 ml of DMEM containing 5% FCS and subjected to three cycles of freeze and thaw using liquid nitrogen and a thermo block set to 37 °C. Samples were then centrifuged at 10,000 × *g* for 10 min at 4 °C to remove cell debris, and cell-associated infectivity was determined by TCID_50_ assay.

### Metabolic radiolabeling of proteins and immunoprecipitation.

Huh-7.5 cells were transfected with indicated HCV genomes. After 24 h, cells were washed with PBS, starved in methionine-free medium for 60 min, and incubated for 6 h in methionine-free DMEM supplemented with 100 μCi/ml of Express Protein labeling mix (Perkin Elmer, http://www.perkinelmer.com/). Cells were lysed either directly or washed several times and incubated in complete DMEM for time periods given in the text. Cell lysates were prepared by using NPB (50 mM, Tris-Cl [pH 7.5], 150 mM NaCl, 1% Nonidet P-40, 1% sodium deoxycholate, and 0.1% SDS), lysates were cleared by centrifugation at 13,800 × *g* for 15 min at 4 °C. The cleared lysates were used for immunoprecipitation using E2-specific antibody AP33 [44]. Immuncomplexes were resolved by denaturing SDS-PAGE and detected by autoradiography.

### Quantitative detection of HCV core protein by ELISA.

HCV C protein was quantified using the Trak-C Core ELISA (Ortho Clinical Diagnostics, http://www.orthoclinical.com/) as recently described [21].

### Amino acid sequence analyses.

Sequence analyses were performed using the euHCVdb database Website facilities at Institute de Biologie et Chimie des Protéines (IBCP) (http://euhcvdb.ibcp.fr/) [45]. Multiple sequence alignments and amino acid conservation were carried out with the ClustalW program using default parameters [46]. The repertoire of residues at each amino acid position and their frequencies observed in natural sequence variants were computed by using a program developed by our laboratory (C. Combet, F. Dorkelt, F. Penin, and G. Deleage, unpublished data).

## Supporting Information

Figure S1 Model of JFH1-Derived p7 This model was constructed by using the secondary structure of a GT 1b p7 variant recently solved by nuclear magnetic resonance as a template (F. Penin et al., unpublished data) and Swiss Model server facilities (http://www.expasy.ch/swissmod/). The structure backbone is represented as ribbon and only the side chains of amino acids mutated in this study and of the residues likely involved in inter-helix interactions are shown. Color code is as follows: Ser, Thr, Asn, and Glu (yellow); Cys (green); His (cyan); Arg and Lys (blue); Glu (red); Trp (magenta); Tyr (purple); any other hydrophobic residue (gray). This figure was generated with Rasmol 2.7 program. The phospholipid bilayer is schematically represented.
(74 KB PDF)

### Accession Numbers

The GenBank (http://www.ncbi.nlm.nih.gov/Genbank/) accession numbers for the HCV genomes used in this study are JFH1 (AB047639), J6CF (AF177036), and Con1 (AJ238799).

## Abbreviations

BVDV: bovine viral diarrhea virus
E1 and E2: envelope glycoprotein 1 and 2
GT: genotype
HCV: hepatitis C virus
TCID_50_: tissue culture 50% infective dose
TM: transmembrane

## References

[ppat-0030103-b001] Simmonds P, Bukh J, Combet C, Deleage G, Enomoto N (2005). Consensus proposals for a unified system of nomenclature of hepatitis C virus genotypes. Hepatology.

[ppat-0030103-b002] Hoofnagle JH (2002). Course and outcome of hepatitis C. Hepatology.

[ppat-0030103-b003] Manns MP, Wedemeyer H, Cornberg M (2006). Treating viral hepatitis C: Efficacy, side effects, and complications. Gut.

[ppat-0030103-b004] Bartenschlager R, Frese M, Pietschmann T (2004). Novel insights into hepatitis C virus replication and persistence. Adv Virus Res.

[ppat-0030103-b005] Penin F, Dubuisson J, Rey FA, Moradpour D, Pawlotsky JM (2004). Structural biology of hepatitis C virus. Hepatology.

[ppat-0030103-b006] Sakai A, Claire MS, Faulk K, Govindarajan S, Emerson SU (2003). The p7 polypeptide of hepatitis C virus is critical for infectivity and contains functionally important genotype-specific sequences. Proc Natl Acad Sci U S A.

[ppat-0030103-b007] Lohmann V, Körner F, Koch JO, Herian U, Theilmann L (1999). Replication of subgenomic hepatitis C virus RNAs in a hepatoma cell line. Science.

[ppat-0030103-b008] Carrere-Kremer S, Montpellier-Pala C, Cocquerel L, Wychowski C, Penin F (2002). Subcellular localization and topology of the p7 polypeptide of hepatitis C virus. J Virol.

[ppat-0030103-b009] Patargias G, Zitzmann N, Dwek R, Fischer WB (2006). Protein-protein interactions: Modeling the hepatitis C virus ion channel p7. J Med Chem.

[ppat-0030103-b010] Griffin SD, Beales LP, Clarke DS, Worsfold O, Evans SD (2003). The p7 protein of hepatitis C virus forms an ion channel that is blocked by the antiviral drug, amantadine. FEBS Lett.

[ppat-0030103-b011] Clarke D, Griffin S, Beales L, Gelais CS, Burgess S (2006). Evidence for the formation of a heptameric ion channel complex by the hepatitis C virus p7 protein in vitro. J Biol Chem.

[ppat-0030103-b012] Pavlovic D, Neville DC, Argaud O, Blumberg B, Dwek RA (2003). The hepatitis C virus p7 protein forms an ion channel that is inhibited by long-alkyl-chain iminosugar derivatives. Proc Natl Acad Sci U S A.

[ppat-0030103-b013] Premkumar A, Wilson L, Ewart GD, Gage PW (2004). Cation-selective ion channels formed by p7 of hepatitis C virus are blocked by hexamethylene amiloride. FEBS Lett.

[ppat-0030103-b014] Gonzalez ME, Carrasco L (2003). Viroporins. FEBS Lett.

[ppat-0030103-b015] Lindenbach BD, Evans MJ, Syder AJ, Wölk B, Tellinghuisen TL (2005). Complete replication of hepatitis C virus in cell culture. Science.

[ppat-0030103-b016] Wakita T, Pietschmann T, Kato T, Date T, Miyamoto M (2005). Production of infectious hepatitis C virus in tissue culture from a cloned viral genome. Nat Med.

[ppat-0030103-b017] Zhong J, Gastaminza P, Cheng G, Kapadia S, Kato T (2005). Robust hepatitis C virus infection in vitro. Proc Natl Acad Sci U S A.

[ppat-0030103-b018] Yi M, Villanueva RA, Thomas DL, Wakita T, Lemon SM (2006). Production of infectious genotype 1a hepatitis C virus (Hutchinson strain) in cultured human hepatoma cells. Proc Natl Acad Sci U S A.

[ppat-0030103-b019] Pietschmann T, Kaul A, Koutsoudakis G, Shavinskaya A, Kallis S (2006). Construction and characterization of infectious intragenotypic and intergenotypic hepatitis C virus chimeras. Proc Natl Acad Sci U S A.

[ppat-0030103-b020] Tscherne DM, Jones CT, Evans MJ, Lindenbach BD, McKeating JA (2006). Time- and temperature-dependent activation of hepatitis C virus for low-pH-triggered entry. J Virol.

[ppat-0030103-b021] Koutsoudakis G, Kaul A, Steinmann E, Kallis S, Lohmann V (2006). Characterization of the early steps of hepatitis C virus infection by using luciferase reporter viruses. J Virol.

[ppat-0030103-b022] Griffin SD, Harvey R, Clarke DS, Barclay WS, Harris M (2004). A conserved basic loop in hepatitis C virus p7 protein is required for amantadine-sensitive ion channel activity in mammalian cells but is dispensable for localization to mitochondria. J Gen Virol.

[ppat-0030103-b023] Shuck K, Lamb RA, Pinto LH (2000). Analysis of the pore structure of the influenza A virus M(2) ion channel by the substituted-cysteine accessibility method. J Virol.

[ppat-0030103-b024] Park SH, Mrse AA, Nevzorov AA, Mesleh MF, Oblatt-Montal M (2003). Three-dimensional structure of the channel-forming trans-membrane domain of virus protein “u” (Vpu) from HIV-1. J Mol Biol.

[ppat-0030103-b025] Kelly ML, Cook JA, Brown-Augsburger P, Heinz BA, Smith MC (2003). Demonstrating the intrinsic ion channel activity of virally encoded proteins. FEBS Lett.

[ppat-0030103-b026] Scarselli E, Ansuini H, Cerino R, Roccasecca RM, Acali S (2002). The human scavenger receptor class B type I is a novel candidate receptor for the hepatitis C virus. EMBO J.

[ppat-0030103-b027] Bartosch B, Vitelli A, Granier C, Goujon C, Dubuisson J (2003). Cell entry of hepatitis C virus requires a set of co-receptors that include the CD81 tetraspanin and the SR-B1 scavenger receptor. J Biol Chem.

[ppat-0030103-b028] Gastaminza P, Kapadia SB, Chisari FV (2006). Differential biophysical properties of infectious intracellular and secreted hepatitis C virus particles. J Virol.

[ppat-0030103-b029] Cocquerel L, Wychowski C, Minner F, Penin F, Dubuisson J (2000). Charged residues in the transmembrane domains of hepatitis C virus glycoproteins play a major role in the processing, subcellular localization, and assembly of these envelope proteins. J Virol.

[ppat-0030103-b030] Liljestrom P, Lusa S, Huylebroeck D, Garoff H (1991). In vitro mutagenesis of a full-length cDNA clone of Semliki Forest virus: The small 6,000-molecular-weight membrane protein modulates virus release. J Virol.

[ppat-0030103-b031] Loewy A, Smyth J, von Bonsdorff CH, Liljestrom P, Schlesinger MJ (1995). The 6-kilodalton membrane protein of Semliki Forest virus is involved in the budding process. J Virol.

[ppat-0030103-b032] Klimkait T, Strebel K, Hoggan MD, Martin MA, Orenstein JM (1990). The human immunodeficiency virus type 1-specific protein Vpu is required for efficient virus maturation and release. J Virol.

[ppat-0030103-b033] Strebel K, Klimkait T, Maldarelli F, Martin MA (1989). Molecular and biochemical analyses of human immunodeficiency virus type 1 Vpu protein. J Virol.

[ppat-0030103-b034] Strebel K, Klimkait T, Martin MA (1988). A novel gene of HIV-1, *vpu,* and its 16-kilodalton product. Science.

[ppat-0030103-b035] Yanagi M, St. Claire M, Shapiro M, Emerson SU, Purcell RH (1998). Transcripts of a chimeric cDNA clone of hepatitis C virus genotype 1b are infectious in vivo. Virology.

[ppat-0030103-b036] Harada T, Tautz N, Thiel HJ (2000). E2-p7 region of the bovine viral diarrhea virus polyprotein: Processing and functional studies. J Virol.

[ppat-0030103-b037] Haqshenas G, Dong X, Ewart G, Bowden S, Gowans EJ (2007). A 2a/1b full-length p7 intergenotypic chimeric genome of hepatitis C virus is infectious in vitro. Virology.

[ppat-0030103-b038] Yi M, Ma Y, Yates J, Lemon SM (2007). Compensatory mutations in E1, p7, NS2, and NS3 enhance yields of cell culture-infectious intergenotypic chimeric hepatitis C virus. J Virol.

[ppat-0030103-b039] Blight KJ, McKeating JA, Rice CM (2002). Highly permissive cell lines for subgenomic and genomic hepatitis C virus RNA replication. J Virol.

[ppat-0030103-b040] Friebe P, Boudet J, Simorre JP, Bartenschlager R (2005). Kissing-loop interaction in the 3′ end of the hepatitis C virus genome essential for RNA replication. J Virol.

[ppat-0030103-b041] Koutsoudakis G, Herrmann E, Kallis S, Bartenschlager R, Pietschmann T (2007). The level of CD81 cell-surface expression is a key determinant for productive entry of hepatitis C virus into host cells. J Virol.

[ppat-0030103-b042] Spearman C (1908). The method of “right and wrong cases” (“constant stimuli”) without Gauss's formulae. Br J Clin Psychol.

[ppat-0030103-b043] Kärber G (1931). Beitrag zur kollektiven Behandlung pharmakologischer Reihenversuche. Naunyn Schmiedebergs Arch Exp Pathol Pharmakol.

[ppat-0030103-b044] Clayton RF, Owsianka A, Aitken J, Graham S, Bhella D (2002). Analysis of antigenicity and topology of E2 glycoprotein present on recombinant hepatitis C virus-like particles. J Virol.

[ppat-0030103-b045] Combet C, Charavay C, Grando D, Crisan D, Lopez J (2007). euHCVdb: The European Hepatitis C Virus Database. Nucleic Acids Res.

[ppat-0030103-b046] Thompson JD, Higgins DG, Gibson TJ (1994). ClustalW: Improving the sensitivity of progressive multiple sequence alignment through sequence weighting, position-specific gap penalties, and weight matrix choice. Nucleic Acids Res.

