# Supplementary material for: Hepatitis C Virus p7 Protein Is Crucial for Assembly and Release of Infectious Virions
Source: PLoS Pathog. 2007 Jul 20;3(7):e103. doi: 10.1371/journal.ppat.0030103 (PMC1924870; doi:10.1371/journal.ppat.0030103)
Supplement: Supplementary file 1 [file ppat.0030103.sg001.pdf]

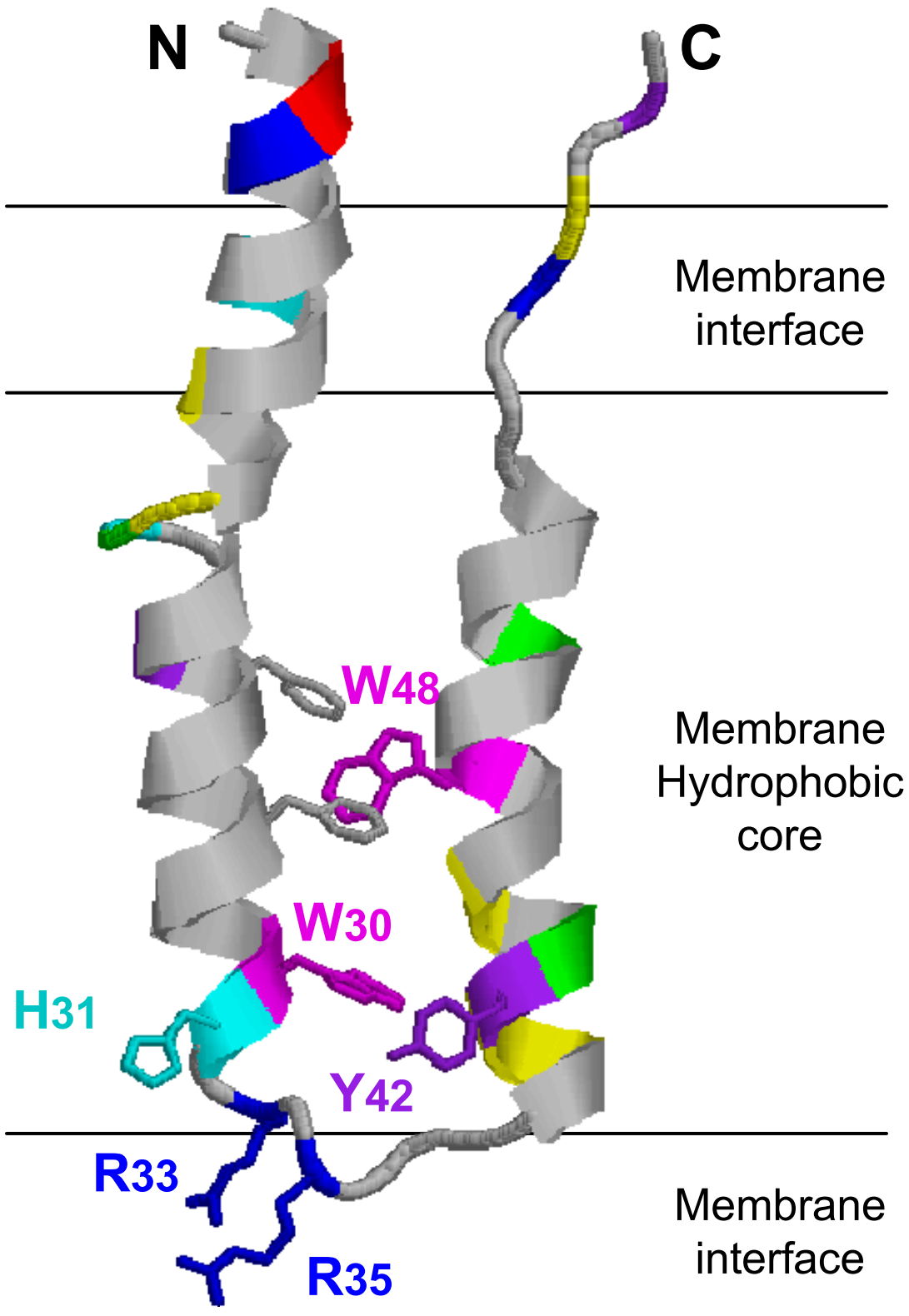
N
C
Membrane
interface
W48
Membrane
Hydrophobic
core
W30
H31
Y42
R33
R35
Membrane
interface

Fig. S1
